# Enhancement and Mechanism of Ergosterol Biosynthesis in Termite Ball Fungus *Athelia termitophila* by Methyl Jasmonate

**DOI:** 10.3390/cimb47030149

**Published:** 2025-02-26

**Authors:** Yong-Gang Fang, Zahid Khan, Fang-Cheng Hu, Xiao-Hong Su, Lian-Xi Xing

**Affiliations:** 1 College of Life Sciences, Northwest University, Xi’an 710069, China; yahoofangyonggang@163.com (Y.-G.F.); sxhnwu@nwu.edu.cn (X.-H.S.); 2 Zoology Department, University of Swabi, Anbar 23560, Khyber Pakhtunkhwa, Pakistan; 3 College of Food Science and Engineering, Northwest A&F University, Xianyang 712100, China; fangchenghu@nwafu.edu.cn; 4 Shaanxi Key Laboratory for Animal Conservation, Northwest University, Xi’an 710069, China

**Keywords:** termite ball fungus, ergosterol, salicylic acid, molecular mechanism, methyl jasmonate

## Abstract

Ergosterol is a component of fungal cell membranes that has physiological functions and applications in drugs, such as anti-inflammatory, anti-tumor, anti-fungal, and other immunosuppressive activities. The fungus *Athelia termitophila*, also known as the termite ball fungus, primarily contains secondary metabolites (like active ingredients) that are similar to ergosterol. To enhance the synthesis of ergosterol and mycelial biomass in termite ball fungus, methyl jasmonate and salicylic acid were used to stimulate the biosynthesis of ergosterol compounds during the growth of TMB mycelium and relative quantitative levels of gene transcripts. The conditions of the inducers were optimized. Under 80 µmol/L MJ incubation conditions, the content of ergosterol compounds in TMB was increased by 2.23-fold compared with the wild-type strain. RT-qPCR results at the transcriptional level of ergosterol synthesis pathway genes showed that MJ significantly induced the expression of *HMGR* (3-Hydroxy-3-Methylglutaryl-Coa Reductase), *HMGS* (3-Hydroxy-3-Methylglutaryl-Coa Synthase), *SE* (Squalene Epoxidase), and *FPS* (Farnesyl Diphosphate Synthase) genes in the ergosterol synthesis pathway. For expression levels at different induction days, we collected 7/10 d and 4/6/8 d samples with similar expression patterns, as well as *SS* (Squalene Synthase)/*FPS* (Farnesyl Diphosphate Synthase), *SE* (Squalene Epoxidase)/*MVD* (Mevalonate Diphosphate Decarboxylase), and *HMGS* (3-Hydroxy-3-Methylglutaryl-Coa Synthase)/*HMGR* (3-Hydroxy-3-Methylglutaryl-Coa Reductase) genes with similar expression patterns, which resulted in gene transcription data during ergosterol content synthesis. The current study provides an effective method to increase the ergosterol contents in termite ball fungus and a new idea for the mechanism of MJ-induced ergosterol compound biosynthesis.

## 1. Introduction

The fungus *Athelia termitophila* (Atheliaceae: Basidiomycota) is a newly discovered fungus found in termite nests over the past decade, and it is also called termite ball fungus (TMB) [1,2]. The mycelium of TMB has been verified to be nutrient-rich and safe through nutritional analysis and safety assessment, making it a potential raw material for food [3]. In addition, our unpublished data suggests that TMB’s mycelium contains ergosterol, triterpenes, cordycepin, adenosine, and other active ingredients that have positive health effects on the human body. However, TMB has the disadvantage of a long growth period in artificial cultivation, which is affected by seasonal and other factors [1]. In contrast, the liquid deep fermentation technique can effectively increase the ability to produce the intended secondary metabolites [4]. However, for industrial development, simple optimization of the medium formulation is insufficient. It has been found that inducers can enhance the synthesis of secondary metabolites by stimulating plant cells to activate many biochemical reactions related to signal transduction pathways [5]. Methyl jasmonate (MJ) is a signaling molecule widely present in plants and has important applications in the regulation of secondary metabolite synthesis in plants and edible mushrooms [6]. Salicylic acid (SA), as an exogenous chemoattractant, is an important signaling molecule in secondary metabolism [7].

It has been demonstrated that the major up-regulated genes were those induced by methyl jasmonate (MJ) when used as an inducer in jasmonate biosynthesis, secondary metabolism, cell wall formation, defense proteins, and stress protection [8]. Salicylic acid (SA) is used as an inducer to activate the expression of key genes in the ergosterol biosynthesis pathway (mevalonate, MVA), which ultimately promotes the accumulation of sterols [9]. The levels of salicylic acid (SA) and methyl jasmonate (MeJA) have been demonstrated to influence cell division and secondary metabolite synthesis [10,11]. Thus, the current investigation examined the circumstances in which MeJA and SA, respectively, stimulated the production of ergosterol in TMB *A. termitophila*. Shi et al. [12] discovered that extended exposure to the inducers or high concentrations resulted in environmental stressors that impacted mycelial development and changed metabolism. Fan et al. [13]. found that methyl jasmonate (MeJA) and exogenous salicylic acid (SA) increased the ergosterol synthesis in *Agaricus* mycelium [13]. The ideal induction conditions were determined by experimental optimization: mycelium was cultivated in liquid culture at 25 °C with either 10.00 μmol/L of SA added on day 6 or 70.00 μmol/L of MeJA added on day 8. After that, both treatments were cultured for two more days. The ergosterol concentrations in the mycelium under these circumstances were 2.35 mg/g for SA and 2.43 mg/g for MeJA. Both Tween-20 and anhydrous ethanol promote the solubility of insoluble drugs in water, which is particularly important for pharmaceutical formulations to form a homogeneous solution [14]. Tween-20 is considered a safe additive in the pharmaceutical and food industries and is widely regarded as a cost-effective co-solvent [15]. Tween-20 is a mild surfactant that has little impact on the natural conformation of proteins, making it ideal for experiments that need to preserve the protein’s natural structure [16]. Anhydrous ethanol is economical and often used as a co-solvent. Proper use of co-solvents improves the effect of the inducer.

Ergosterol is a steroidal compound that forms colorless acicular or flaky crystals with the molecular formula C_28_H_44_O [17]. It exhibits solubility in ethanol, ether, benzene, and trichloromethane and remains insoluble in water [18]. To make vitamin D_2_, ergosterol was dissolved in chloroform, ether, cyclohexane, or other solvents, which were added to a quartz glass flask and exposed to ultraviolet light [19]. Ergosterol is a precursor to vitamin D_2_ and can be used as an intermediate in hormone drugs or to make “cortisone” and “progesterone” [20]. Ergosterol primarily exists in the cell in either a free or bound state, with 90% of these free sterols found in the cell membrane, a crucial component of the fungal cell membrane [21].

Numerous studies have focused on the process of ergosterol biosynthesis, which is mainly accomplished through a complex metabolic pathway involving a variety of enzymes [22,23]. The complex de novo ergosterol biosynthesis involved nearly 30 enzymes, known as Erg proteins [22]. The ergosterol biosynthesis pathway was found in three steps: firstly, mevalonate biosynthesis, followed by farnesyl pyrophosphate biosynthesis, and lastly, ergosterol biosynthesis [21]. Fungi primarily use the mevalonate (MVA) pathway to synthesize ergosterol, a crucial sterol. The structure and function of fungal cell membranes, which include multiple cellular functions, such as membrane fluidity and substance transport, depend on ergosterol, particularly its distribution in yeasts, molds, and certain plants. Multiple essential enzyme genes work in concert to regulate the MVA pathway, a center for ergosterol production, to ensure optimal operation.

Acetyl-coenzyme A (Acetyl-CoA) initiates the MVA pathway, which is catalyzed by a condensing enzyme (*AACT*). Mevalonate synthase (*HMGS*) and mevalonate reductase (*HMGR*) are two enzymes that regulate the amount of mevalonate synthesis [24]. Mevalonate kinase (*PMK*) phosphorylates mevalonate, and mevalonate pyrophosphate decarboxylase (*MVD*) decarboxylates it to isoprenyl pyrophosphate (*IPP*), a crucial precursor for the synthesis of ergosterol [25]. The dynamic equilibrium between IPP and dimethylallyl pyrophosphate (DMAPP) is controlled by isoprene pyrophosphate isomerase (*IDI*). The immediate precursor for the production of squalene, farnesyl pyrophosphate (*FPP*), is then produced from *IPP* and DMAPP by farnesyl pyrophosphate synthase (*FPS*) [26]. Squalene synthase (*SS*) transforms FPP into squalene at the downstream stage of the route. Squalene epoxidase (*SE*) then oxidizes the resulting squalene to produce 2,3-oxo-squalene, a crucial step in the synthesis of sterols [27]. The pathway’s progression and the buildup of sterols are controlled by the exact expression of these genes.

Nowadays, research on TMB mainly focuses on its parasitism and symbiosis with termites [8,28], but there is no report on its metabolites and the effects of key enzymes or genes of the synthesis pathway. In the present study, we concentrated our research on the roles of methyl jasmonate and salicylic acid in the biosynthesis of ergosterol in TMB. In order to provide a new method for the production of ergosterol compounds bio-synthesized by TMB as a strain, we identified the most effective inducers, their concentrations, the duration required for biosynthesis, and the number of days required for incubation, and examined potential molecular mechanisms of inducer synthesis.

## 2. Materials and Methods

### 2.1. Preparation of Strains and Media Formulation

The termite ball fungus was discovered during a field study in Xi’an by the termite research team of Northwest University and was subsequently isolated and purified, which was also confirmed as the same species as *A. termitophila* found scattered in termite nests in Japan. Information on biological preservation is available in CCTCC M 2018446.

To culture TMB, a Comprehensive Potato Solid Medium (CPDA) was obtained from a mixture of potato extract powder (24 g/L), potassium dihydrogen phosphate (3 g/L), magnesium sulfate (1.5 g/L), and agar (18 g/L). Comprehensive Potato Liquid Medium (CPDB) was obtained from a mixture of potato extract powder (24 g/L), potassium dihydrogen phosphate (3 g/L), and magnesium sulfate (1.5 g/L).

### 2.2. Prepare the Liquid Strain and Revitalize the Strain

The TMB was inoculated into potato dextrose agar (PDA) and then stored in a refrigerator at a constant temperature of 4 °C. Before use, the original strain was inoculated into fresh solid medium (CPDA) and put into a 24 °C constant temperature incubator (Cool Incubator i-CUBE (HOT & COOL) Japan, AS ONE Corporation in Osaka, Japan) for growth for 6–8 days.

The activated TMB was inoculated into potato liquid medium (CPDB) by punching four agar blocks with a hole punch and then cultured in a constant temperature shaker incubator (HZQ-F160A, Suzhou, China) at 24 °C and 160 r/min for 7 days as a first-class seed liquid for reserve.

### 2.3. TMB Mycelium Collection and Biomass Determination

After several days of incubation, the shake flasks were removed and placed in a Brinell’s funnel (Bel-Art H14602-0000, Nanjing, China) containing four layers of gauze, then washed with distilled water several times during the filtration process and pumped with a vacuum filtration pump (SHB-III, Cangzhou, China), after which the resulting cake was placed in a 55 °C blast dryer (DHG-9053, Shanghai, China) to dry until its weight did not change, i.e., the mycelial biomass.

The mycelium filaments were ground and passed through an 80-mesh sieve, and the collected mycelial powder was stored at 4 °C for spare use. All independent experiments were repeated in three parallels.

### 2.4. Mycelial Ergosterol Extraction and Determination and Analysis

The previously mentioned spare mycelium powder was taken, transferred to a closed test tube, and utilized anhydrous ethanol as a solvent for ultrasonic extraction, centrifugation, filtration, and recovery of the supernatant, which served as the sample solution for testing. The standard ergosterol was extracted using ethanol as the solvent, followed by centrifugation and filtration, and then the supernatant was collected as the standard solution. The standard solution and the sample solution to be tested were placed in the appropriate test tubes, and then 1 mL of the solution was pipetted and filtered through a 0.45 μm disposable filter into a sample bottle for HPLC (Agilent HPLC 1260, Shanghai, China) determination. HPLC chromatographic conditions: DIKMAC18 (250 mm × 4.6 mm, 5 µm); mobile phase: 100% methanol; flow rate: 1.0 mL/min; column temperature: 20 °C; injection volume: 10 µL; detection wavelength: 282 nm. The standard curve can be used to determine the ergosterol content in TMB after making a standard curve using ergosterol standards [29].

### 2.5. Effect of Different Concentrations of MJ and SA on Ergosterol-Producing Synthesis by TMB

Various inducers were chosen to study ergosterol in TMB to determine the best inducer and concentration. According to a previous study by Kurowska et al. [1], SA and MJ were separately dissolved in 80 μmol/L anhydrous ethanol as a co-solvent and then filtered through a 0.45 μm filter membrane for sterilization. The medium was initially sterilized, followed by the addition of the SA and MJ solutions. The experimental procedure will the final concentrations of MJ were 0, 10, 20, 50, 80, 100, and 150 μmol/L, and the final concentrations of salicylic acid were 0, 50, 100, 150, 200, 250, and 300 μmol/L. Equal volumes of ethanol treatment were given to the controls. The goal was to determine the best concentration of the optimal inducer. All experiments were performed in triplicate, and the resulting values are shown as mean ± standard deviation.

### 2.6. *Effect of Various Co-Solvents on Ergosterol Levels in TMB After Dissolving MJ*

Anhydrous ethanol and Tween-20 were chosen as the co-solvents of methyl jasmonate for the experiments; these were filtered through a 0.45 um filter membrane to remove the omnibacteria, and the methyl jasmonate solution was added to the cultures at the start of the experiments after the omnibacteria were removed. This resulted in the final concentration of methyl jasmonate being 0, 10, 20, 50, 80, 100, and 150 μ mol/L; the control was given an equivalent volume of ethanol treatment. Getting the ideal co-solvent was the goal. Values are presented as mean ± standard deviation, and all experiments were run in triplicate.

### 2.7. Effect of Different Addition Times on the Ergosterol Content of TMB

The ergosterol-producing by TMB was investigated under the optimum concentration of MJ obtained by dissolving in anhydrous ethanol as a solvent, removing the bacteria by filtration through a 0.45 µm filter membrane, and adding the MJ solution to the cultures at the beginning of the experiment on the 0th day. Different days (7, 8, 9, 10, 11, 12, and 13) were selected for the incubation. All the experiments were performed in triplicate, and the values are shown as the mean ± standard deviation of ergosterol compounds.

### 2.8. Impact of Induction Time Variation on the Ergosterol Content of TMB

To study the effect of the optimal concentration of MJ on the yield of ergosterol-producing compounds by the TMB, the MJ was dissolved in anhydrous ethanol as a solvent, and the MJ solution was added to the deep liquid fermentation cultures of TMB on days 0, 2, 4, 6, 7, 8, and 10, respectively. We added an optimized concentration of MJ in 80 μmol/L, total incubated it for 12 days, and eliminated the sterility of the cultures by filtering them through a 0.45 µm nylon filter membrane. All the experiments were performed in triplicate, and the values are shown as mean ± standard deviation of ergosterol compounds.

### 2.9. Measurement of Gene Transcription Levels by qRT-PCR

Quantitative real-time polymerase chain reaction (qRT-PCR) has been widely used for quantitative analysis of gene expression [30]. Internal reference genes for gene expression quantification by qRT-PCR are critical for normalizing differential gene expression between samples, but their expression often varies greatly depending on tissues, treatments, and growth condition [31]. The expression experiment was performed using a 96-well plate on a CFX Connect machine (BIO-RAD, Hercules, CA, USA) with the One Step SYBR Prime Script PLUS RT-PCR kit (TaKaRa, TaKaRa code: DRR096A). The final RT reaction volume was 20 μL, which consisted of 10 mL 2X SYBR Premix Ex Taq II (TaKaRa, Kusatsu, Japan), 0.4 μL of forward primer, and 0.4 μL reverse primer. The PCR cycling condition was as follows: 95 °C for 30 s, 40 cycles of 95 °C for 10 s, and 60 °C for 30 s. Three technical replicates were used for each sample, and averages of three biological replicates were counted. Dettman et al. [32] investigated some genes as internal reference genes for qRT-PCR in ginseng at various growth stages and in different tissue genes. Wang et al. [33] conducted a similar study in different tissues of ginseng and ginseng seedlings under heat stress. In this study, we extracted total RNA from the mycelium from the previous experiments and then used a reverse-transcription premixed kit to reverse-transcribe the extracted total RNA into cDNA. First, the cDNA was then considered to be the template for qRT-PCR determination. Onward, the Premix Pro Taq HS qPCR Kit (AccurateBiotechnology (Hunan) Co., Ltd., Changsha, Hunan Province, China) was used for RT-PCR. We screened gene sequences from transcriptome data mainly including *AACT* (Acetyl-CoA acetyltransferase), *MVD* (mevalonate diphosphate decarboxylase), *IDI* (Isopentenyl diphosphosphate isomerase), *FPS* (Farnesyl Pyrophosphate Synthase), *HMGR* (3-hydroxy-3-methylglutaryl-CoA reductase), *HMGS* (3-Hydroxy-3-methylglutaryl-CoA synthase), *SS* (Sterol Synthase), *SE* (Sterol 14α-demethylase), and *PMK* (Phosphomevalonate kinase), where the 18S RNA (18S ribosomal RNA gene) gene serves as the housekeeping gene. Primers were designed using NCBI (Table 1), and the relative expression of each gene was assessed using gene-specific primers.

The relative gene expression was standardized by determining the changes in ergosterol content in TMB. Under MJ treatment, the 18S RNA gene of TMB was selected, and the relative stability of the 18S RNA gene was verified by the correlation analysis between ergosterol content and the expression of genes related to ergosterol biosynthesis. For each gene, the reference sample’s expression level was 1.0, and the results under other conditions were expressed as fold changes in mRNA levels relative to the reference sample. By correlation analysis of ergosterol content with the expression of ergosterol biosynthesis-related genes, qRT-PCR reactions were performed in a LineGene real-time fluorescent quantitative PCR detection system and calculated using the 2^−ΔΔCt^ method [34].

### 2.10. Organization and Analysis of Data

All experiments were repeated three times independently, and data were expressed as mean ± standard deviation. The resultant data were analyzed using SPSS 20.0 (IBM, Armonk, NY, USA), variations in means of treatments were counted by one-way ANOVA, and significant difference analysis was performed by Duncan’s new complex polarity method. All data were in triplicate (mean ± standard deviation). Different letters indicate significant differences (*p* < 0.05, according to the Duncan test). Correlation, cluster analyses, and plots were performed using Origin 2020 (OriginLab Corporation, Northampton, MA, USA).

## 3. Results

### 3.1. Effects of Varying Inducer Concentrations on TMB’s Ergosterol Components and Mycelium Biomass

**Figure 1:**
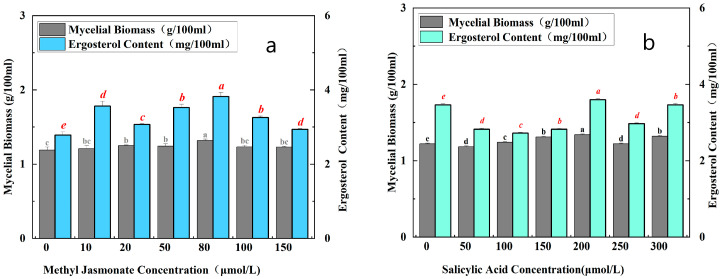
Results of TMB effects stimulated by different concentrations of MJ (**a**) and SA (**b**). Different letters such as ‘a’ and ‘b’ are used to indicate statistically significant differences between treatment groups. No SA or MJ was added to any of the control groups. All fermentations were carried out at 24 °C for 7 days.

As the concentration of MJ increased from 10 μmol/L to 80 μmol/L, the content of ergosterol compounds increased gradually, compared with the increase in mycelial biomass, and the ergosterol content reached a peak at the concentration of MJ of 80 μmol/L, 1.37 times that of the control group. This concentration significantly increased the synthesis of ergosterol compounds induced by MJ. In addition, mycelial biomass also reached the highest level. As the concentration of MJ continued to increase, the content of ergosterol compounds began to decrease slowly, along with the mycelial biomass. Thus, high concentrations of MJ are detrimental to mycelial growth (Figure 1a). As the concentration of SA increased from 50 µmol/L to 200 μmol/L, the content of ergosterol compounds changed, but the change in mycelial biomass was not significant, and the ergosterol content peaked at 200 μmol/L, 1.03 times higher than the control (Figure 1b). SA triggered the enhancement in ergosterol synthesis, coinciding with the peak mycelial biomass. However, further increases in SA concentration led to a gradual decline in both ergosterol content and mycelial biomass, suggesting that elevated concentrations are detrimental to mycelial growth.

### 3.2. Effect of MJ Solubilization by Different Solvents on Mycelial Growth and Ergosterol

**Figure 2:**
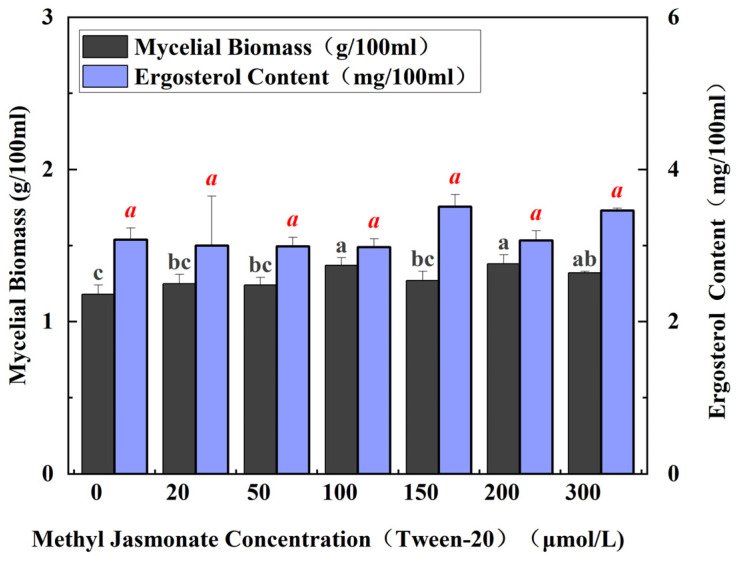
Mycelial biomass and ergosterol compound content under different MJ concentrations. No MJ was added to any of the control groups. All fermentations were carried out at 24 °C for 7 days. Different letters such as ‘a’ and ‘b’ are used to indicate statistically significant differences between treatment groups. No SA or MJ was added to any of the control groups. All fermentations were carried out at 24 °C for 7 days.

However, the induction effect of MJ varies greatly depending on the additives. After the above experiments, the best inducer was MJ at a concentration of 80 μmol/L, and the maximum yield was 1.37-fold higher than that of the untreated control group. However, no study was conducted on the co-solvents, and the selection of different co-solvents may change the culture environment and thus affect the ergosterol compound content. The above experiments used anhydrous ethanol as a co-solvent. In this study, Tween-20 and anhydrous ethanol were chosen as the co-solvents of MJ to observe their dynamic changes in mycelial growth and ergosterol content. The co-solvent of Tween-20 alone was added to the culture medium. The study revealed that Tween-20 significantly stimulated mycelial growth and ergosterol biosynthesis. As the concentration of MJ increased from 20 μmol/L to 150 μmol/L, the ergosterol compound content increased slowly. No increase was observed in mycelial biomass by comparison at 150 μmol/L. At 150 μmol/L MJ concentration, the ergosterol content reached a peak 1.14 times higher than that of the control, suggesting that the synthesis of ergosterol compounds triggered by MJ solubilized by Tween-20 at this concentration increased most significantly. Additionally, the mycelial biomass reached its highest level (Figure 2). As the concentration of MJ continued to increase, the content of ergosterol compounds began to slowly decrease, as well as mycelial biomass; therefore, dissolving high concentrations of MJ with Tween-20 was detrimental to mycelial growth. In contrast, anhydrous ethanol is more effective as a co-solvent.

### 3.3. Effects of Different Incubation Times on Mycelial Growth and Ergosterol Compound Content of TMB

**Figure 3:**
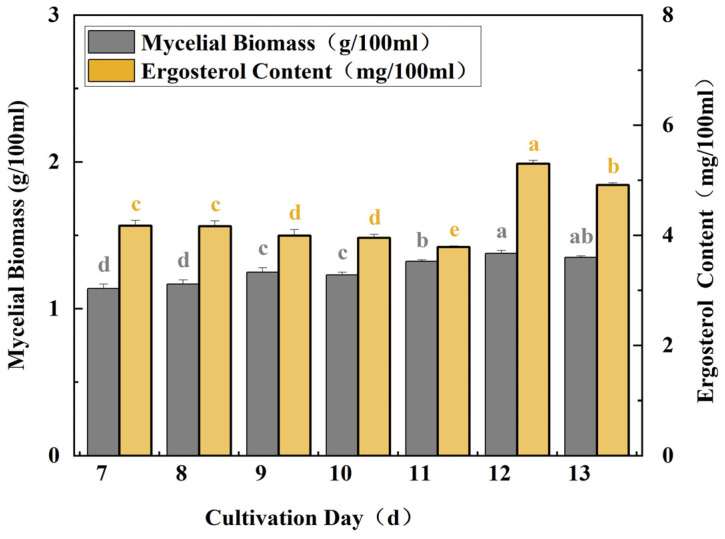
Mycelial biomass and ergosterol compound content of different culture times. All fermentations were carried out at 24 °C for 7 days.

MJ dissolved in anhydrous ethanol was added on the 0th day, and the final concentration was 80 µmol/L. The results of the ergosterol content and mycelial biomass of the MJ-treated strips at different times showed that the ergosterol content increased slowly as the incubation period increased from 9 d to 11 d, and the mycelial biomass was synchronous with the trend of ergosterol synthesis. However, ergosterol content increased rapidly from day 11 to day 12. The ergosterol compound content and mycelial biomass peaked on day 12, which was 1.26 times higher than the control group. With the extension of incubation time, ergosterol content and mycelial biomass began to decrease significantly (Figure 3).

### 3.4. Effect of MJ Addition at Different Times on Ergosterol Composition During Fermentation of TMB

**Figure 4:**
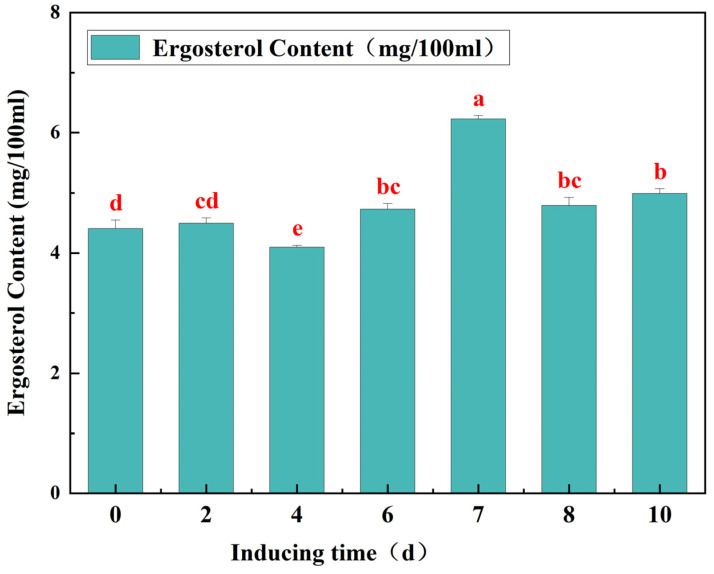
Effect of different days on the production of ergosterol compounds by TMB under the stimulation of 80 μmol/L MJ.

In order to investigate the effect of different addition times of MJ on ergosterol biosynthesis in TMB, we added MJ on the 0th, 2nd, 4th, 6th, 7th, 8th, and 10th days to the oscillating culture of TMB. As time went on, the ergosterol level rose until it reached 5.505 mg/100 mL when 80 µmol/L MJ dissolved in anhydrous ethanol was added on the seventh day. This level was 1.25 times higher than that of the control group. At subsequent addition times, both ergosterol compound content and mycelial biomass began to decrease significantly (Figure 4).

### 3.5. Expression of Related Genes Under Different Concentrations of MJ Treatments

**Figure 5:**
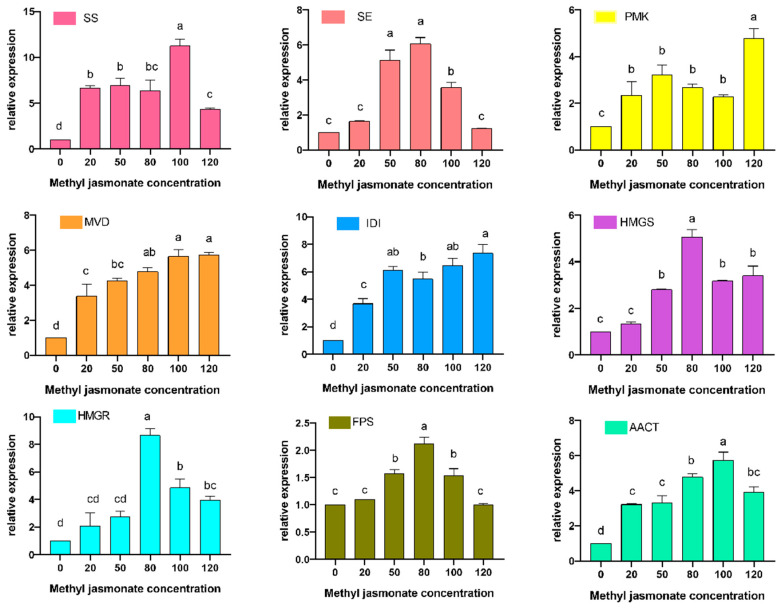
Relative expression of genes on the pathway in TMB under MJ at different concentrations without inducer application to the control treatments in the above experiments. All fermentations were carried out at 24 °C for 7 d.

Transcriptome sequencing was applied to the treated samples to screen the expression data of genes associated with the ergosterol synthesis pathway, and a total of 10 genes were investigated. RT-qPCR was used to confirm that the genes involved in the ergosterol synthesis pathway were expressed in the same samples (Figure 5). In this experiment, the relative expression of genes was mainly analyzed by the 2^−ΔΔCt^ method. In order to improve the reliability of the data, six sets of replicates were set up between different samples, the caretaker genes were set as 18s genes, and the internal reference genes were verified to be available by the laboratory. Post-experimental data showed that most of the genes were up-regulated after MJ induction. Several genes had the highest transcript levels, and these were also the optimal concentrations for MJ. For example, *HMGR*, *HMGS*, *SE*, and *FPS* showed the highest transcript levels at 80 μmol/L, which were 8.68, 5.07, 6.06, and 4.78-fold higher than the control samples, respectively. However, there were some genes whose highest expression concentration was not at 80 μmol/L. For example, the highest expression concentration of *PMK* (*Phosphomevalonate kinase*) was 120 μmol/L, which was 4.79 times higher than that of the control samples, while the highest expression concentration of *AACT* (*Acetyl-CoA acetyltransferase*) was 100 μmol/L, which was 5.07 times higher than that of the control samples.

### 3.6. Analysis of Ergosterol Content and Gene Correlation Under MJ Treatment

**Figure 6:**
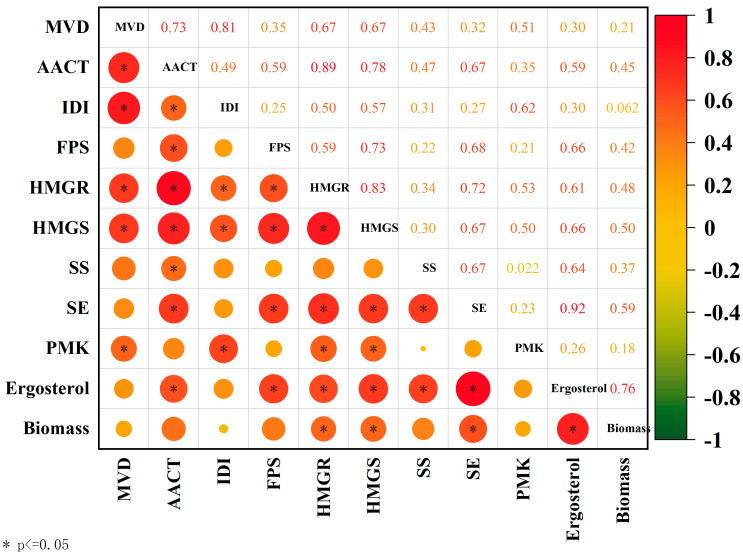
Intergroup correlation analysis of ergosterol compounds, mycelial biomass, and ergosterol compound synthesis genes.

To further investigate the correlation among the mycelial biomass, ergosterol content, and transcript levels of several ergosterol biosynthesis genes under MJ treatment, we performed Spearman correlation analysis using Origin software version 2020 (OriginLab Corporation, Northampton, MA, USA). The results indicated that ergosterol content had a highly significant positive correlation with mycelial biomass, with a correlation coefficient of 0.76 (*p* < 0.01). The transcript-level analysis of ergosterol content and ergosterol biosynthesis genes revealed that the relative transcript levels of *HMGR*, *HMGS*, *SE*, and *FPS* genes were significantly and positively correlated with ergosterol content. The correlation coefficients were 0.61, 0.66, 0.92, and 0.66 (*p* < 0.001), respectively (Figure 6). Therefore, it was concluded that these genes may play an important role in the biosynthesis of ergosterol compounds.

Conversely, a highly significant positive correlation was found between the *HMGR* gene and the *HMGS* and *SE* genes (*p* < 0.001) when the four highly significant genes linked to ergosterol synthesis were compared. The *HMGS* gene showed a highly significant positive correlation with the *SE* gene, with a correlation coefficient of 0.67 (*p* < 0.001), and the *FPS* gene showed a highly significant positive correlation with *HMGR*, while the *HMGS* and *SE* genes showed a highly significant positive correlation with correlation coefficients of 0.59, 0.73, and 0.68 (*p* < 0.001), respectively.

### 3.7. Effect of Key Genes and Time Dynamics Under MJ Treatment Conditions

**Figure 7:**
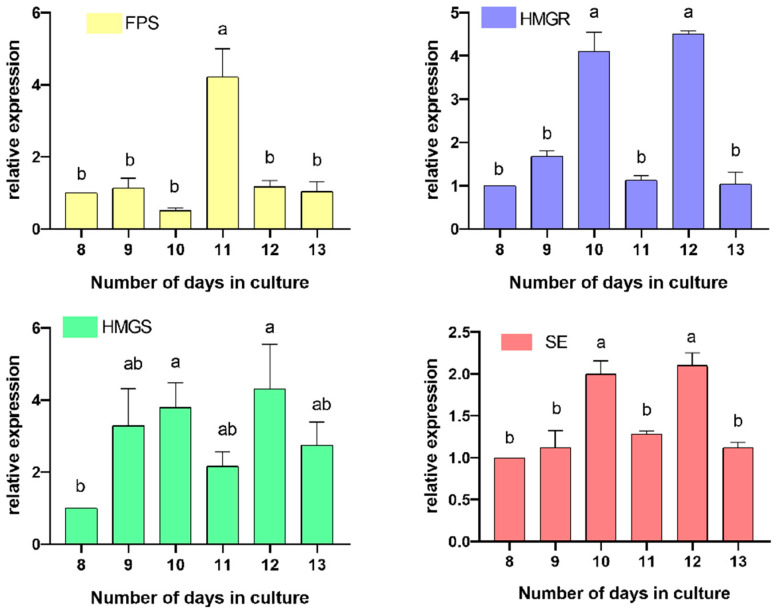
Relative expression of genes on the pathway in TMB under the optimal concentration of MJ treatment. In graphs, letters (e.g., a, b, ab) are often used to indicate statistically significant differences. These letters are the results of subsequent multiple comparison tests and are used to show whether differences between treatment groups or time points are statistically significant. The letter above each bar graph indicates whether the gene expression level at that time point is significantly different compared to other time points. For example, in the *FPS* expression graph, the bar on day 11 is labelled ‘a’ while the other time points are labelled ‘b’, which indicates that the *FPS* expression level on day 11 is significantly different compared to the other time points.

A high correlation (*p* < 0.001) was found between metabolite accumulation and gene expression. The transcript levels of the four key genes from the correlation analysis were studied further by qRT-PCR to see how the transcript levels changed over time. The transcript levels of ergosterol biosynthesis genes (*HMGR*, *SE*, *FPS*, and *HMGS*) were dynamically found. The results showed that on the 12th day under MJ stimulation, *HMGR*, *SE*, and *HMGS* reached the highest transcript levels of 4.51-fold, 2.11-fold, and 4.31-fold of those of the control groups; and on the 11th day, the transcript level of *FPS* reached the highest level, about 4.22-fold of that of the control group (Figure 7).

### 3.8. Clustered Expression Analysis of Genes Related to Different Periods in Response to MJ

**Figure 8:**
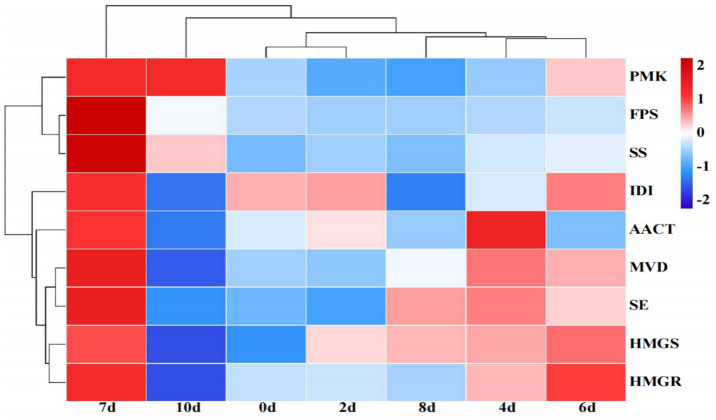
As shown in the TMB Heatmap, MJ caused changes in genes involved in the ergosterol synthesis pathway at different times.

The transcriptional data of the genes involved in different periods of MJ stimulation were analyzed using qRT-PCR clustering, and a heat map showed the group. The results indicate that four main groups, namely the 0 d and 2 d groups; 7 d groups; 4 d, 6 d, and 8 d groups; and 10 d groups, exhibited a gradual response to MJ stimulation over time. Genes showed high expression levels at 4 d, 6 d, and 7 d, and low expression levels at the start of the fermentation period (2 d) and the later stage of the fermentation period (10 d). This pattern of gene expression aligns with the ergosterol synthesis situation. Furthermore, we grouped some biosynthetic genes with comparable expression patterns, revealing three primary groups: *FPS* and *SS* were grouped together with similar expression patterns, and these two genes were ergosterol synthesis pathway genes; *MVD* and *SE* were grouped with similar expression patterns, and these two genes were ergosterol metabolism pathway genes; *HMGR* and *HMGS* were grouped with similar expression patterns. The gene clustering results were largely consistent with the positions of ergosterol synthesis pathway genes, which later confirmed the reliability of the clustering analysis results (Figure 8).

## 4. Discussion

Ergosterol, a sterol compound structurally similar to cholesterol, is commercially produced through yeast fermentation or extraction from fungal mycelium. It serves as a precursor for vitamin D2 and steroid hormone drugs, and its derivatives exhibit significant antitumor and anti-HIV activities [27]. As a natural steroid, ergosterol possesses antioxidant, anti-inflammatory, anticancer, and antiviral properties [29]. Importantly, the ergosterol biosynthetic pathway is a critical target for developing antifungal drugs, as it is essential for fungal growth and survival.

By upregulating the expression of genes encoding important enzymes, methyl jasmonate (MJ) has been demonstrated to encourage the buildup of secondary metabolites [1]. Through a variety of physiological and biochemical processes, MJ has a favorable impact on plant metabolites and a variety of impacts on secondary metabolites in plant tissue culture systems [30]. Geng et al. [31] discovered that the accumulation of jasmonates (JA) and 4′-deoxyflavones in the roots of the fungus *Scutellaria baicalensis* is associated with senescence and pathogen infection; genes associated with pathogen resistance and senescence have been examined. On the fifteenth day of incubation in a ginseng cell suspension culture investigation, 200 μmol/L MJ was introduced. After 8 days, the ginseng cells achieved their highest total ginsenoside yield, with a 4-fold increase in the content of Rb1, Rb2, Rc, and Rd. Specifically, the content of the ginsenoside monomer Rb1 increased 4-fold, while Rb2, Rc, and Rd increased slightly. The content of Rg1 and Re increased 2.3-fold and 3.0-fold, respectively [32]. In *Ganoderma lucidum*, MeJA significantly increased ganoderic acid production by activating genes in the mevalonate pathway [11]. Similarly, in *Agaricus* species, MeJA boosted ergosterol synthesis by upregulating *HMGR* and *SE* [13]. These studies align with our findings, where MeJA induced the expression of *HMGR*, *HMGS*, *SE*, and *FPS*, directly linking its role to ergosterol biosynthesis in fungi.

In this study, we used MJ to stimulate the synthesis of ergosterol compounds in *Athelia termitophila* (TMB), increase biomass, and investigate changes in gene transcription levels at the molecular level. The dose of inducer and the length of fermentation time significantly impact metabolite yield and cell growth in a particular culture system [33,34]. We treated TMB with varying concentrations of salicylic acid (SA, 0–300 μmol/L) and MJ (0–150 μmol/L). The best conditions for biosynthesis of ergosterol and mycelial biomass were achieved when TMB was treated with 80 μmol/L MJ, with an induction period of 7 days, followed by a fermentation period of 12 days. We found that MJ significantly regulates ergosterol production during the culture of TMB. Through RNA sequencing (RNA-seq) and analyses of gene metabolism, we revealed the mechanisms by which MJ enhances ergosterol production in TMB through various pathways. Based on these findings, we also identified key genes that play crucial roles in improving ergosterol yield.

The results of this investigation provide important light on the regulatory processes governing TMB’s ergosterol synthesis. Using MJ as a stimulant raises biomass and improves the yield of ergosterol molecules, indicating possible uses in large-scale manufacturing. To further maximize ergosterol production, genetic engineering may target the known essential genes involved in ergosterol biosynthesis.

Future studies could investigate how MJ interacts with other inducers or environmental factors to optimize ergosterol yield. Furthermore, we can use advanced genetic and metabolic engineering techniques to develop strains with enhanced ergosterol synthesis capabilities. This research lays a solid foundation for creating sustainable and effective methods of ergosterol production, which could have significant implications for industrial and pharmaceutical applications.

## 5. Conclusions

The results of this experiment showed that we found the best solvent and its optimal concentration to promote the growth of TMB, which increased the yield of ergosterol compounds. In addition, we analyzed the number of incubation days and induction time and obtained the optimal concentration of 80 μmol/L. The incubation at days 12 and 7 resulted in the content of ergosterol of 6.229 ± 0.06 mg/100 mL. The qRT-PCR analysis identified *HMGS*, *HMGR*, *FPS*, and *SE* as the key genes associated with ergosterol synthesis. Cluster integration dynamically detected and analyzed their spatiotemporal transcript levels. The results will help enhance the yield of ergosterol compounds in TMB even further and will aid our understanding of the functioning of key enzymes and biosynthesis steps in the ergosterol biosynthetic pathway in TTAbleMB.

## Figures and Tables

**Table 1:** Primer synthesis sequence.

Primer Name	Sequence (5′-3′)
SE-132-F	CGGTCGTGCTCGTGAAGGG
SE-132-R	GGAGGATGTGCGTCGTTATATGC
FPS-187-F	CATCGGCAAACAAACTGGCA
FPS-187-R	GAGAACAAGGAAGGCACCGA
IDI-138-F	GTGACACCCAATGAGAACGAA
IDI-138-R	CCACCAGCCGAACAGGAA
PMK-200-F	CGGATGATGCTTGCTGATGT
PMK-200-R	GCTGTGAGCGTGTAAGTGCC
HMGS-153-F	GTCTTCGATGGAGTGTCTAAGGG
HMGS-153-R	ACCGATAGATTTTGGGTCGATGT
32 HMGR-127-F	ACCAACCAACAACGGCGA
32 HMGR-127-R	GCCCTTCACACCGAGCAT
33 HMGR-200-F	CTTGGCTCGCCGTGTTTC
33 HMGR-200-R	TTACTGCTCTTCATTTCGCCTATT
MVD-191-F	TATGGTTGAACGGCAAGGTAGA
MVD-191-R	TGATGCGGAAGATGCGAGA
SS-182-F	TGGATACCATTGAGGACGACA
SS-182-R	TGGAGAAAGGCGGTTGACT
AACT-221-F	CTATCAAGGGCAAGAAGGGTG
AACT-221-R	GGAGATGACTTTGGCAAGAGGTT
18S rna-164-F	ACATCCGCCATCCATTCC
18S rna-164-R	TCGCTGCCCATCACCATA

## Data Availability

The original contributions presented in this study are included in the article; further inquiries can be directed to the corresponding author.

## References

[1] Kurowska M.M., Daszkowska-Golec A., Gajecka M., Kościelniak P., Bierza W., Szarejko I. (2020). Methyl Jasmonate Affects Photosynthesis Efficiency, Expression of HvTIP Genes and Nitrogen Homeostasis in Barley. Int. J. Mol. Sci..

[2] Matsuura K. (2006). Termite-egg mimicry by a sclerotium-forming fungus. Proc. R. Soc. B Biol. Sci..

[3] Zhao J. (2020). Rejuvenation preservation and determination of active substances of *Termitococcus* spp.. Master’s Thesis.

[4] Vassilev N., Eichler-Löbermann B., Flor-Peregrin E., Martos V., Reyes A., Vassileva M. (2017). Production of a potential liquid plant bio-stimulant by immobilized Piriformospora indica in repeated-batch fermentation process. AMB Express.

[5] Ozenirler S., Erkan G., Gülbahar O., Bostankolu O., Ozbaş Demırel O., Bilgihan A., Akyol G. (2011). Serum levels of advanced oxidation protein products, malonyldialdehyde, and total radical trapping antioxidant parameter in patients with chronic hepatitis C. Turk. J. Gastroenterol..

[6] Fedderwitz F., Nordlander G., Ninkovic V., Björklund N. (2016). Effects of jasmonate-induced resistance in conifer plants on the feeding behaviour of a bark-chewing insect, *Hylobius abietis*. J. Pest Sci..

[7] Wang X., Sun J., Wang S., Sun T., Zou L. (2023). Salicylic acid promotes terpenoid synthesis in the fungi *Sanghuangporus baumii*. Microb. Biotechnol..

[8] Cheong J.-J., Choi Y.D. (2003). Methyl jasmonate as a vital substance in plants. Trends Genet..

[9] Ye J., Mao D., Cheng S., Zhang X., Tan J., Zheng J., Xu F. (2020). Comparative transcriptome analysis reveals the potential stimulatory mechanism of terpene trilactone biosynthesis by exogenous salicylic acid in Ginkgo biloba. Ind. Crops Prod..

[10] Hu Y.-H., Yu Y.-T., Piao C.-H., Liu J.-M., Yu H.-S. (2011). Methyl jasmonate- and salicylic acid-induced d-chiro-inositol production in suspension cultures of buckwheat (*Fagopyrum esculentum*). Plant Cell Tissue Organ Cult. (PCTOC).

[11] Ren A., Qin L., Shi L., Dong X., Mu D.S., Li Y.X., Zhao M.W. (2010). Methyl jasmonate induces ganoderic acid biosynthesis in the basidiomycetous fungus Ganoderma lucidum. Bioresour. Technol..

[12] Shi L., Tan Y., Sun Z., Ren A., Zhu J., Zhao M. (2019). Exogenous Salicylic Acid (SA) Promotes the Accumulation of Biomass and Flavonoid Content in *Phellinus igniarius* (Agaricomycetes). Int. J. Med. Mushrooms.

[13] Fan X.Z., Yao F., Yin Z.M. (2021). Exogenous induction of ergosterol synthesis in Agaricus blazei. Mod. Food Sci. Technol..

[14] Kong P., Hong C. (2024). Evaluation of 1021Bp, a close relative of Pseudomonas eucalypticola, for potential of plant growth promotion, fungal pathogen suppression and boxwood blight control. BMC Microbiol..

[15] Knoch H., Ulbrich M.H., Mittag J.J., Buske J., Garidel P., Heerklotz H. (2021). Complex Micellization Behavior of the Polysorbates Tween 20 and Tween 80. Mol Pharm.

[16] Chou D.K., Krishnamurthy R., Randolph T.W., Carpenter J.F., Manning M.C. (2005). Effects of Tween 20^®^ and Tween 80^®^ on the Stability of Albutropin During Agitation. J. Pharm. Sci..

[17] Merdivan S., Lindequist U. (2017). Ergosterol Peroxide: A Mushroom-Derived Compound with Promising Biological Activities-A Review. Int. J. Med. Mushrooms.

[18] Klemptner R.L., Sherwood J.S., Tugizimana F., Dubery I.A., Piater L.A. (2014). Ergosterol, an orphan fungal microbe-associated molecular pattern (MAMP). Mol. Plant Pathol..

[19] Lasunción M.A., Martín-Sánchez C., Canfrán-Duque A., Busto R. (2012). Post-lanosterol biosynthesis of cholesterol and cancer. Curr. Opin. Pharmacol..

[20] Zhao J., Lin W., Ma X., Lu Q., Ma X., Bian G., Jiang L. (2010). The protein kinase Hal5p is the high-copy suppressor of lithium-sensitive mutations of genes involved in the sporulation and meiosis as well as the ergosterol biosynthesis in Saccharomyces cerevisiae. Genomics.

[21] Hu Z., He B., Ma L., Sun Y., Niu Y., Zeng B. (2017). Recent Advances in Ergosterol Biosynthesis and Regulation Mechanisms in Saccharomyces cerevisiae. Indian J. Microbiol..

[22] Sokolov S.S., Trushina N.I., Severin F.F., Knorre D.A. (2019). Ergosterol Turnover in Yeast: An Interplay between Biosynthesis and Transport. Biochemistry.

[23] Oliaro-Bosso S., Balliano G., Viola F., Ferrante T. (2016). Difference in the late ergosterol biosynthesis between yeast spheroplasts and intact cells. Acta Biochim. Pol..

[24] Sayari M., van der Nest M.A., Steenkamp E.T., Rahimlou S., Hammerbacher A., Wingfield B.D. (2021). Characterization of the Ergosterol Biosynthesis Pathway in Ceratocystidaceae. J. Fungi.

[25] Dinday S., Ghosh S. (2023). Recent advances in triterpenoid pathway elucidation and engineering. Biotechnol. Adv..

[26] Xu W., Yao J., Liu L., Ma X., Li W., Sun X., Wang Y. (2019). Improving squalene production by enhancing the NADPH/NADP^+^ ratio, modifying the isoprenoid-feeding module and blocking the menaquinone pathway in *Escherichia coli*. Biotechnol. Biofuels.

[27] Micera M., Botto A., Geddo F., Antoniotti S., Bertea C.M., Levi R., Gallo M.P., Querio G. (2020). Squalene: More than a Step toward Sterols. Antioxidants.

[28] Matsuura K., Yashiro T., Shimizu K., Tatsumi S., Tamura T. (2009). Cuckoo Fungus Mimics Termite Eggs by Producing the Cellulose-Digesting Enzyme β-Glucosidase. Curr. Biol..

[29] Zhang Z., Wu D., Wang Y.Y., Yang Y., Feng J., Li W., Chen W.-C., Zhang J.S. (2021). Optimization of liquid fermentation process of ergosterol produced by *Hericium erinaceus*. Mycosystema.

[30] Udvardi M.K., Czechowski T., Scheible W.-R. (2008). Eleven Golden Rules of Quantitative RT-PCR. Plant Cell.

[31] Geng D., Jiang M., Dong H., Wang R., Lu H., Liu W., Guo L., Huang L., Xiao W. (2023). MeJA regulates the accumulation of baicalein and other 4′-hydroxyflavones during the hollowed root development in *Scutellaria baicalensis*. Front. Plant Sci..

[32] Dettman R.W., Liu J., Wang Q., Sun M., Zhu L., Yang M., Zhao Y. (2014). Selection of Reference Genes for Quantitative Real-Time PCR Normalization in Panax ginseng at Different Stages of Growth and in Different Organs. PLoS ONE.

[33] Wang M., Lu S. (2015). Validation of Suitable Reference Genes for Quantitative Gene Expression Analysis in Panax ginseng. Front. Plant Sci..

[34] Zhou H., Li L., Wang K., Zhao M., Li S., Jiang Y., Zhu L., Chen J., Wang Y., Sun C. (2019). Selection and validation of reference genes desirable for gene expression analysis by qRT-PCR in MeJA-treated ginseng hairy roots. PLoS ONE.

